# The Foreign Body That Wasn’t

**DOI:** 10.1016/j.acepjo.2026.100539

**Published:** 2026-11-02

**Authors:** Jacob Gibbs, Greer Guthrie, Yagnaram Ravichandran-Cupp, Inna Kaminecki

**Affiliations:** Department of Emergency Medicine, Texas Tech University Health Sciences Center, Lubbock, Texas, USA

**Keywords:** acute appendicitis, appendicolith, pediatric emergency medicine, foreign body ingestion, autism spectrum disorder, atypical presentation, diagnostic imaging

## 1 Case Presentation

A 5-year-old nonverbal male with autism spectrum disorder and congenital fourth nerve palsy presented with acute distress, restlessness, and recurrent vomiting. His mother reported he suddenly “did not look right,” and appeared uncomfortable. Earlier, he was evaluated at an outside emergency department, received ceftriaxone for otitis media, and was discharged. Symptoms persisted with multiple episodes of emesis. On arrival, he was afebrile and hemodynamically stable. Physical examination was limited by developmental status and revealed no peritoneal findings. Laboratory studies demonstrated leukocytosis (22,500 cells/μL) with neutrophilic predominance. Abdominal radiography demonstrated a radiopaque density over the right iliac wing (Fig 1), concerning for an ingested foreign body with obstruction at the ileocolic junction. Given diagnostic uncertainty, computed tomography abdomen/pelvis was obtained and demonstrated a 10-mm appendicolith within an enlarged appendix with periappendiceal fat stranding (Fig 2). The patient was admitted for appendectomy.

**Figure 1:**
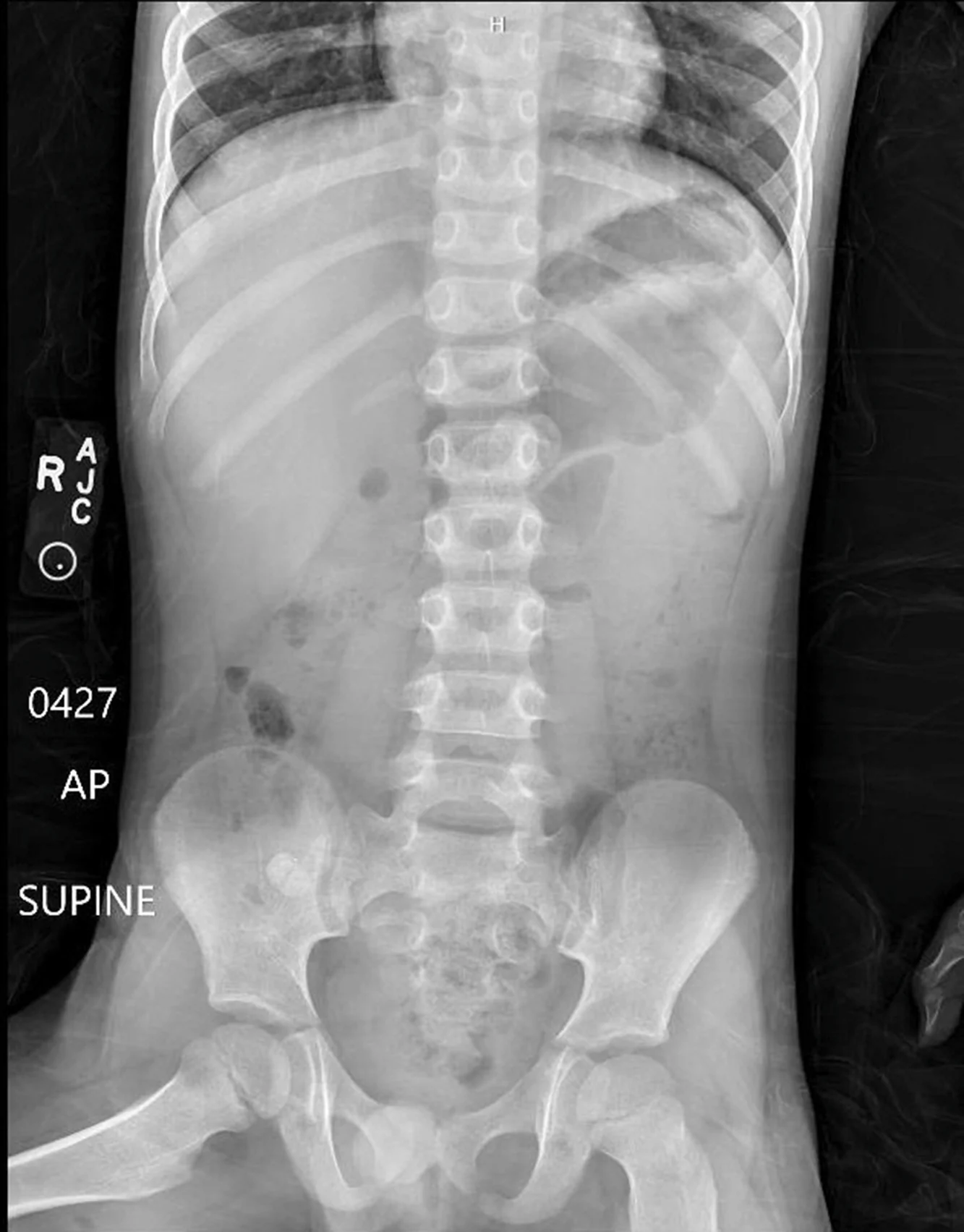
Abdominal radiograph demonstrating a radiopaque density projected over the right lower abdomen, initially concerning for an ingested foreign body.

**Figure 2:**
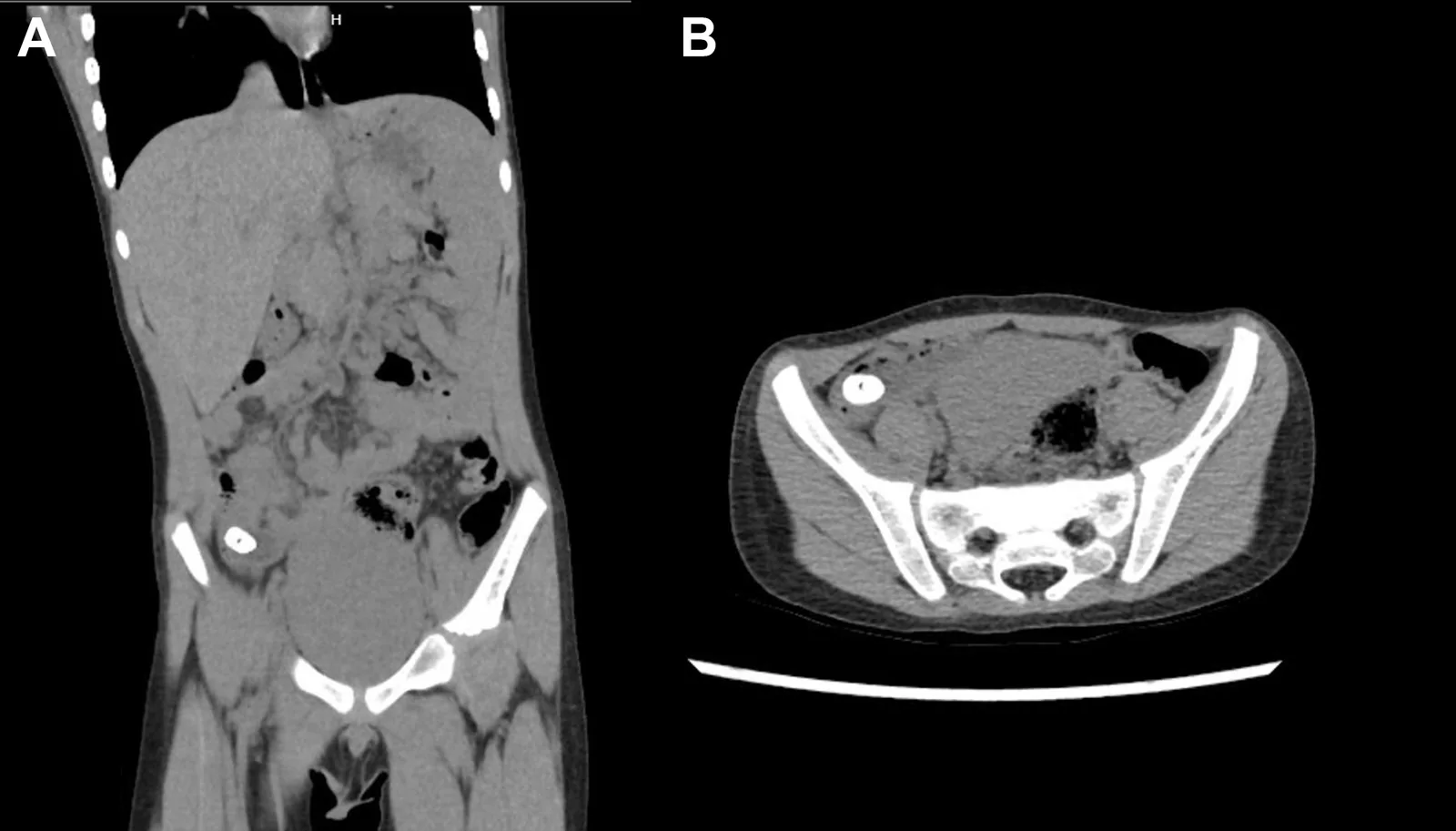
A, Computed tomography of the abdomen and pelvis demonstrating a large appendicolith at the base of the appendix with associated appendiceal enlargement and periappendiceal inflammatory fat stranding consistent with acute appendicitis. B, Cross-sectional computed tomography of the abdomen and pelvis demonstrating a large appendicolith (arrow) at the base of the appendix consistent with acute appendicitis.

## 2 Diagnosis: Acute Appendicitis With Appendicolith

Acute appendicitis remains the most common pediatric surgical emergency and may be difficult to diagnose in children with communication barriers.^1^, ^2^ In nonverbal patients, caregiver observations and behavioral changes may provide the most important diagnostic clues. Appendicoliths are associated with increased disease severity and a higher risk of perforation.^3^, ^4^ Occasionally visible on plain radiographs, they may mimic ingested foreign bodies. Persistent clinical concern despite a nondiagnostic examination should prompt advanced imaging.^5^ This case highlights the importance of maintaining a broad differential diagnosis and recognizing that a radiopaque density on abdominal radiography may represent an appendicolith, particularly in nonverbal pediatric patients presenting with unexplained vomiting and distress.

## Funding and Support

By *JACEP Open* policy, all authors are required to disclose any and all commercial, financial, and other relationships in any way related to the subject of this article as per ICMJE conflict of interest guidelines (see www.icmje.org). The authors have stated that no such relationships exist.

## Conflict of Interest

All authors have affirmed they have no conflicts of interest to declare.
